# European consensus statement on diagnosis and treatment of adult ADHD: The European Network Adult ADHD

**DOI:** 10.1186/1471-244X-10-67

**Published:** 2010-09-03

**Authors:** Sandra JJ Kooij, Susanne Bejerot, Andrew Blackwell, Herve Caci, Miquel Casas-Brugué, Pieter J Carpentier, Dan Edvinsson, John Fayyad, Karin Foeken, Michael Fitzgerald, Veronique Gaillac, Ylva Ginsberg, Chantal Henry, Johanna Krause, Michael B Lensing, Iris Manor, Helmut Niederhofer, Carlos Nunes-Filipe, Martin D Ohlmeier, Pierre Oswald, Stefano Pallanti, Artemios Pehlivanidis, Josep A Ramos-Quiroga, Maria Rastam, Doris Ryffel-Rawak, Steven Stes, Philip Asherson

**Affiliations:** 1 PsyQ, psycho medische programma's, Department Adult ADHD, Carel Reinierszkade 197, Den Haag, The Netherlands; 2 Department of Clinical Neuroscience, Karolinksa Institutet, Section Psychiatry, St. Goran, Stockholm, Sweden; 3 University Department of Psychiatry, Addenbrookes Hospital, Cambridge, UK; 4 Pediatric Department, Hôpitaux Pédiatriques CHU-Lenval, 06200 Nice, France; 5 Servicio de Psiquiatria, Hospital Universitari Vall d' Hebron, Universidad Autonoma de Barcelona, Barcelona, Spain; 6 Reinier van Arkel Groep, Postbus 70058, 5201 DZ 's-Hertogenbosch, The Netherlands; 7 Department of Neuroscience/Psychiatri Ulleråker, MK 75, S-750 17 Uppsala, Sweden; 8 Institute of Development, Research, Advocacy and Applied Care (IDRAAC), Department of Psychiatry and Clinical Psychology, St George Hospital University Medical Centre, Balamand University, Beirut, Lebanon; 9 Centre des Consultations, Institut A Tzanck, Mougins, France; 10 Department of Psychiatry, Trinity College Dublin (TCD), Dublin 2, Ireland; 11 Clinique des Maladies Mentales et de l'Encéphale (CMME), Sainte Anne Hospital Paris, France; 12 Affektiva mottagningen, M 59, Psykiatri Sydväst, 141 86 Stockholm, Sweden; 13 Département de Psychiatrie Adulte, Unité Lescure, CH Charles Perrens, Bordeaux, France; 14 Private clinic for psychiatry and psychotherapy, 11a Schillerstrasse, Ottobrunn, Germany; 15 Department of Child Neurology, Oslo University Hospital, Ullevaal, Oslo, Norway; 16 Geha Mental Health Center, Petach-Tiqva, Sackler Faculty of Medicine, Tel-Aviv University, Israel; 17 Department of Child Psychiatry, Regional Hospital of Bolzano, Via Guncina, Bolzano, Italy; 18 Faculty of Medical Sciences, New University of Lisbon, Campo dos Mártires da Pátria, 1169-056 Lisboa, Portugal; 19 Department of Psychiatry, Social Psychiatry and Psychotherapy, Hannover Medical School, Germany; 20 Department of Psychiatry, Brugmann University Hospital, Université Libre de Bruxelles, Brussels, Belgium; 21 Department of Neurosciences, Florence University, Florence, Italy; 22 Department of Psychiatry, National and Kapodistrian University of Athens Medical School, Eginition Hospital, Athens, Greece; 23 Programa Integral del Déficit de Atención en el Adulto (P.I.D.A.A), Servei de Psiquiatria, Hospital Universitari Vall d'Hebron, Barcelona, Spain; 24 Department of Clinical Sciences, Lund, Child and Adolescent Psychiatry, Lund University, Sweden; 25 Johanniterstraße 1, CH-3047 Bremgarten, Bern, Switzerland; 26 ADHD Program, University Psychiatric Center, Catholic University Leuven, Kortenberg, Belgium; 27 MRC Social Genetic and Developmental Psychiatry, Institute of Psychiatry, Kings College London, London, UK

## Abstract

**Background:**

Attention deficit hyperactivity disorder (ADHD) is among the most common psychiatric disorders of childhood that persists into adulthood in the majority of cases. The evidence on persistence poses several difficulties for adult psychiatry considering the lack of expertise for diagnostic assessment, limited treatment options and patient facilities across Europe.

**Methods:**

The European Network Adult ADHD, founded in 2003, aims to increase awareness of this disorder and improve knowledge and patient care for adults with ADHD across Europe. This Consensus Statement is one of the actions taken by the European Network Adult ADHD in order to support the clinician with research evidence and clinical experience from 18 European countries in which ADHD in adults is recognised and treated.

**Results:**

Besides information on the genetics and neurobiology of ADHD, three major questions are addressed in this statement: (1) What is the clinical picture of ADHD in adults? (2) How can ADHD in adults be properly diagnosed? (3) How should ADHD in adults be effectively treated?

**Conclusions:**

ADHD often presents as an impairing lifelong condition in adults, yet it is currently underdiagnosed and treated in many European countries, leading to ineffective treatment and higher costs of illness. Expertise in diagnostic assessment and treatment of ADHD in adults must increase in psychiatry. Instruments for screening and diagnosis of ADHD in adults are available and appropriate treatments exist, although more research is needed in this age group.

## Review

### The European Network Adult ADHD

The European Network Adult ADHD was founded in 2003 and includes 40 professionals from 18 countries across Europe with an interest in and experience of ADHD in adults http://www.adult-adhd.net. This independent expert panel was set up to help improve the diagnosis and management of ADHD in adults throughout Europe. It is the opinion of the panel that today, due to lack of recognition and misunderstanding about the disorder and the use of appropriate, stimulant or non-stimulant medication, to control symptoms of ADHD, many adults with ADHD are misdiagnosed and are often prevented from receiving effective treatments. This may lead to unnecessary suffering for individual patients, their families and work colleagues.

### Objectives of consensus statement

The objectives of this consensus statement are to increase awareness of the following: (1) That ADHD often presents as an impairing lifelong condition in adults, yet is currently underdiagnosed and treated in many European countries; (2) That instruments for screening and diagnosis of ADHD in adults are available; (3) That appropriate treatments exist.

Three major questions are addressed in this statement: (1) What is the clinical picture of ADHD in adults? (2) How can ADHD in adults be properly diagnosed? (3) How should ADHD in adults be effectively treated?

### Methodology

This document is the result of three meetings between 2003 and 2009 in which the need for communication and a consensus statement in Europe were identified. A formally prepared document with consensus statements agreed by experts within the group was reviewed and discussed. Following this process the final document was circulated for written approval by all members of the European Network. For the assembly of the document, reviews and randomised controlled trials in adults were identified from Medline, Embase and the Cochrane Database, as well as from reviews on ADHD in children and cross-referencing and identification by the participating experts. Details of the clinical presentation of adults with ADHD were further informed by clinical expertise. This consensus statement is written for specialists in psychiatry but is also intended to increase the understanding of the disorder and to facilitate referral for diagnosis, treatment and follow-up from primary health care physicians and other health care providers.

## Background

ADHD is among the most common psychiatric disorders in childhood with well established diagnostic and treatment services available throughout most of Europe. Until recently, the disorder was considered by many to resolve during adolescence and young adulthood with little or no continued impact in adult life [1], although descriptions of the adult condition appeared in the psychiatric literature from 1976 onwards [2]. However current evidence indicates that in the majority of cases ADHD persists into adult life where it is associated with a range of clinical and psychosocial impairments. Numerous follow-up studies of children with ADHD show that the disorder persists during adolescence and adulthood in around two-thirds of individuals [3-11] either as the full blown disorder or in 'partial remission' with persistence of some symptoms associated with continued clinical and psychosocial impairments. In the meta-analysis of these data from Faraone and colleagues it was concluded that about 15% retain the full diagnosis by age 25 years, with a further 50% in partial remission [12], indicating that around two-thirds of children with ADHD continue to have impairing levels of ADHD symptoms as adults.

Although in some cases the symptoms of ADHD may appear to diminish during adolescence, this may not be the case relative to controls and does not mean that functioning is unimpaired. In a follow-up study of 119 boys of 19 years of age with childhood onset ADHD, symptom levels seemed to be lower than in childhood but 90% still did not function well [13]. Importantly, although symptoms levels appear to reduce as people grow older there are parallel changes among control groups; so that significant case-control differences are retained [14].

In a study from the World Health Organisation Mental Health Survey, it was found that childhood predictors of adult ADHD included the combined subtype of ADHD in childhood, symptom severity, the presence of comorbid depression, high rates of other comorbidities, social adversity and parental psychopathology [3]; while Biederman had previously reported family history of ADHD, psychosocial adversity and comorbidity with conduct, mood and anxiety disorders to be predictors of persistence [15]. Nevertheless, all forms of ADHD are known to persist into adulthood including ADHD with predominantly inattentive symptoms and ADHD associated with milder levels of impairment and comorbidity.

The prevalence of ADHD in adults estimated from epidemiological studies is in the range of 2-5% [16-19]. Persistent forms of ADHD are thought to have a higher familial loading than ADHD that does not persist, with increased rates of ADHD among the parents and siblings of those with persistent ADHD and high rates of ADHD among the offspring of parents with ADHD [20]. Twin and adoption studies indicate that the familiality of ADHD symptoms results from genetic factors rather than shared environmental risks, providing a further rationale for considering ADHD as a lifetime condition [21]. ADHD occurs in around 10-20% of people with common mental health problems according to epidemiological and clinical research [22-27]. Further studies show that this rate may be higher in some clinical populations such as those attending forensic, addiction and personality disorder clinics, highlighting the importance of screening within such high risk populations [28].

There is growing recognition of the importance of diagnosing and treating the disorder in parents of children with ADHD [29] since around 20% of parents of children with ADHD will have ADHD themselves [30]. Furthermore, parents with ADHD may have difficulty in implementing parent training strategies for the treatment of behavioural problems in their offspring. Since the recognition of ADHD is relatively recent throughout much of Europe there are in addition many adults with ADHD who were never diagnosed or treated for ADHD when they were children [31]. Recent national guidelines now recommend that ADHD should be recognised and appropriately treated throughout the lifespan [32-34]. Despite this, across much of Europe many professionals working in adult mental health services remain unaware that ADHD frequently persists into adult life and remain uninformed about the clinical presentation and the consequences of ADHD across the lifespan.

Another reason for underdiagnosis and treatment of ADHD in adults is the age-dependent change in the presentation of ADHD symptoms. The more overtly impairing symptoms in childhood, hyperactivity and impulsivity, often become less obvious in adulthood, shifting the problem to more subtle symptoms such as inner restlessness, inattention, disorganisation and to impairment in behaviours related to executive functioning; and this may lead to discontinuation of treatment when they are still required [13,31,35-39]. Additional reasons for underdiagnosis of ADHD include the frequent presence of comorbid psychiatric syndromes, which in clinical practice may be identified as the primary or only diagnosis. Finally, stigma and myths continue to surround the condition and its treatment, particularly with stimulant medication [40,41].

### Stigma in general and among professionals

ADHD is an established disorder in childhood with child and adolescent mental health or paediatric services for ADHD available across most of Europe. Yet adult services for people with ADHD remain relatively scarce despite strong evidence for the benefits of diagnosing and treating ADHD in adults (reviewed in [32]). There are still many professionals that are unsure of the diagnosis and the appropriate use of ADHD medications in adult mental health. Some continue to express fears about treating a 'non-existent disease' or causing drug addiction with stimulant medication, despite evidence to the contrary [42]. The reasons for this are likely to be based on the historical perception of ADHD as a disorder that is restricted to childhood and the continued presence of stigma and clinical mythology that surrounds the disorder and its treatment; and the traditional separation of adult from child psychiatry. What is clear is that there remains a gulf in the perception of the disorder between those working in paediatric and child and adolescent mental health services and those working in adult mental health, that cannot be explained on the basis of validated evidence based information [43].

Stigma related to the term ADHD is one component of the problem that nearly always arises in the context of lack of awareness or understanding of available data. Within the mental health profession stigma is further associated with the restricted regulatory status in many countries for most of the medications that treat ADHD in adults, but has other reasons as well. ADHD in adults remains a disorder which is poorly understood and where an 'emotional burden' is attached to the term especially among professionals who have not traditionally been involved in the diagnosis or treatment of ADHD [42,44,45]. People suffering from ADHD are often stereotyped as lazy, bad or aggressive, or considered to have a behavioural or special needs problem rather than a mental health disorder that requires treatment [44]. The diagnosis may also be overlooked because ADHD is a highly symptomatic disorder and those less familiar with the onset, course, psychopathology and comorbidities associated with the disorder may mistake ADHD for other common mental health problems such as mood or personality disorders.

While increasing awareness and availability of accurate information is a high priority, many education programs for primary care physicians lack a component for ADHD so that a high percentage remain unaware of the way that ADHD affects people beyond the childhood years [45]. Furthermore, education about adult ADHD has not been included in most college programs for medical and psychology students, as well as training of professionals in adult mental health. Education programs therefore need to target all stages of professional development, from students through to primary and secondary care physicians and psychologists, to ensure appropriate early recognition, diagnosis and treatment are provided. Referral to specialist clinics should be possible where secondary care physicians lack sufficient training for more complex cases.

In terms of treatment, stimulants are by far the best studied and most effective treatment for ADHD across the lifespan, yet their use in some parts of Europe remains controversial in children and more widely across Europe in adults. The recent National Institute for Health and Clinical Excellence (NICE) guidelines from the UK describes the situation in which a drug treatment is considered safe to give to children but not safe to give to adults as an "anomaly" [32]. The NICE guidelines have been pivotal in the UK by providing national guidance for the development of clinical services for adults with ADHD and the recommendation of stimulants as the usual first line treatment. As a consequence many new clinics are being established and increasing numbers of adults are provided with effective treatment in the form of stimulants, despite the lack of formal recognition by regulatory bodies for use of medicines. The current lack of licensed indications for the use of stimulants in adults in most European countries (but not in the US) is not supported by available data, but rather results from the historical focus on ADHD as a child disorder, commercial considerations by pharmaceutical companies and caution from regulators: a situation that may be revised in the next 1-2 years in Europe as several formulations of methylphenidate and dexamphetamine are being put forward for registration. However, a recent safety review of the use of methylphenidate from the European Medicines Agency, which restricts its recommendation to children over 6 years of age and adolescents and does not mention use in adults, has led to methylphenidate no longer being licensed for use in countries such as Norway.

### Genes, environment and neurobiology

Family, twin and adoption studies show that ADHD is a familial disorder with high heritability, indicating that a significant genetic component influences risk for the disorder [46-54]. Environmental factors are also likely to play a role either as main causal factors in a few cases [55] or by interaction with genetic risks.

Family studies indicate a risk to first degree relatives of 4 to 10 fold the population rate, with prevalence among first degree relatives in the range of 20-50% [20]. Data from numerous twin studies of parent and teacher rated ADHD in children and adolescents indicate an average heritability (the variance explained by additive genetic factors) of around 76% [56], indicating that familial influences on ADHD are largely genetic. Furthermore twin studies that have investigated the continuity of ADHD at various developmental stages indicate that continuity of symptoms across the lifespan is largely the result of shared genetic effects [57-59]. Yet, studies of self-rated ADHD symptoms in adult population twin samples consistently report lower estimates of heritability, in the region of 30-40% from two published [59,60] and one unpublished (Larsson et al., in preparation) study. The reasons for the lower heritability has not been fully investigated, but is likely to arise from the use of self-rating ADHD scales in population twin samples for two main reasons. First there may be a variable level of awareness among individuals when self-rating their own ADHD symptoms, leading to inaccurate ADHD symptom scores; and secondly self-ratings of ADHD symptoms may be confounded by adult onset conditions that generate ADHD-like symptoms, such as anxiety, depression, fatigue and drug and alcohol use. There is however as yet no empirical data to resolve these questions, so we have to conclude that further work is needed to fully understand the extent of genetic influences on ADHD in adults.

Molecular genetic studies in adults are relatively recent but are expected to confirm some genetic associations identified in childhood ADHD samples and find other genes that relate to persistence or remission of ADHD symptoms in adult life. There is a great deal of interest in the mechanisms by which the disorder persists in some individuals and remits in others, since this may identify new targets that prevent progression of the disorder into adult life. One potential mechanism is suggested by the developmental hypothesis of Jeffrey Halperin, which proposed that ADHD is linked to an early-appearing and enduring subcortical dysfunction (weak arousal mechanisms), while symptom remission is dependent on the extent of maturational changes in executive control [61,62]. The emphasis is on the interaction between these two processes, with remission or persistence of ADHD symptoms related to the emerging balance between cortical and sub-cortical function. Whether the processes involved can be neatly separated into sub-cortical versus cortical is uncertain and requires further detailed investigations, however a recent large international study obtained evidence that the same two processes account for 85% and 12% respectively, of the genetic influences on ADHD [63].

Molecular genetic studies of ADHD in children provide direct support for the association of specific genes with ADHD. Genetic variants within or near to the D4 (DRD4) and D5 (DRD5) dopamine receptor genes provide the most consistent findings supported by meta-analysis [64]. Numerous other studies find evidence of association with the dopamine transporter gene (DAT1), the dopamine beta-hydroxylase gene (DBH), the serotonergic transporter (5-HTT), the serotonergic receptor (HTR1B), and the synaptosomal-associated protein, 25 kDa (SNAP-25) [48]. Taken together these candidate gene findings are thought to explain around 3.2% of the variance in ADHD symptoms in children [65]. More recently whole genome association studies have identified novel genes such as CDH13 (a Cadherin gene) as potential risk factors [66] and rare copy number variants that confer higher risks in the order of odds ratios of 2-5, depending on general cognitive ability (Williams et al., reported at the World Congress of Psychiatric Genetics, 2009).

Molecular genetic studies have also turned to the study of ADHD in adults in recent years. Much of the current research coordinated in Europe by Barbara Franke from the Netherlands for the International Multicentre Persistent ADHD Collaboration (IMPACT) group. This collaboration has successfully generated a multi-site sample of around 3,000 patients and is continuing to develop. To date several publications highlight potential associations with ADHD in adults, some but not all of which are shared with genetic association findings in children [67-72].

Environmental factors are also associated with ADHD [73], particularly prenatal risk factors such as exposure to alcohol, nicotine, drugs, high blood pressure and maternal stress during pregnancy, as well as preterm birth and low birth weight [74-76]. Evidence from Romanian adoptees also suggests that severe early deprivation is causally related to ADHD [55]. In some cases the environmental measure may be mediated by genetic effects and may not always implicate environmental exposure as the primary causal factor. For example there is new evidence that prenatal exposure to nicotine may reflect genetic effects rather than the direct toxic effects of nicotine or other constituents of tobacco smoke [77]. Overall it is likely that environmental risk factors play an important role in the aetiology of ADHD and that in many cases the impact of the environment will be modified by genetic factors.

Neurocognitive, neurophysiological and neuroimaging studies suggest that brain dysfunctions are involved in the central components of the syndrome in children and adults [78-82]. Fronto-striatal dysfunction and increased  dopamine transporter density in the striatum have both been reported [83-86] although the finding of increased dopamine transporter density remains a controversial finding, perhaps secondary to drug treatments for ADHD [87,88]. Magnetic resonance imaging (MRI) studies indicate smaller volumes of caudate, corpus callosum, cerebellum and right frontal areas, as well as increased cortical thinning [89-97]. Functional MRI data show differences in brain functioning between ADHD and controls including some studies of drug naïve patients [91,98-103]. Positron emission tomography (PET) shows abnormal cerebral glucose metabolism in prefrontal and premotor areas of the frontal lobe in ADHD adults [104-106]. In addition single photon emission computed tomography studies show  studies show hypoperfusion and hypofunctioning of prefrontal and striatal regions in children and older adults with ADHD compared to controls [107,108]. The immediate and marked response of ADHD symptoms to stimulant medications such as amphetamines and methylphenidate, which increase levels of synaptic dopamine, suggests that the main underlying pathophysiological process may involve deficits or imbalances in catecholaminergic, dopaminergic, and nicotinergic functioning [109-111].

## Clinical Picture of ADHD in adults

### Definition of ADHD

The two most frequently used diagnostic terms to describe the condition in childhood are attention-deficit/hyperactivity disorder (ADHD) and hyperkinetic disorder (HKD). For both definitions the disorder is defined as a clinical syndrome characterised by the presence of developmentally inappropriate levels of inattention, hyperactivity and impulsivity, starting in childhood and leading to impairment. The Diagnostic and Statistical Manual of Mental Disorders (DSM-IV) criteria for ADHD as defined by the American Psychiatric Association are the most widely used criteria and describe three subtypes of ADHD based on the predominant symptom pattern: inattentive type, hyperactive-impulsive type and the combined type [112]. The International Classification of Diseases (ICD-10) criteria for HKD as defined by the World Health Organisation (WHO) are more conservative and define a severe subgroup of people fulfilling the ADHD combined type diagnosis [113]. This results in a lower estimated prevalence of HKD (about 1%) compared to the prevalence estimates of 4-8% for ADHD in childhood [114]. ICD-10 criteria also exclude the presence of common co-occurring disorders including anxiety or depression [115] that are allowed under the DSM-IV definition of ADHD.

The next edition of the DSM (version 5) now in preparation is due to be published in 2013 and the revised ICD (version 11) for 2015. The revised DSMV criteria are expected to follow similar lines to the current criteria with the following expected changes http://www.dsm5.org:

(1) Symptom thresholds: For older adolescents and adults (aged 17 and above) only 4 symptoms in either the inattentive or hyperactive-impulsive domain are required.

(2) The list of hyperactive-impulsive symptoms has been increased to 13 to include 'uncomfortable doing things slowly or carefully', 'is often impatient', 'difficult to resist temptations or opportunities' and 'tends to act without thinking'.

(3) Descriptions of symptom items have been elaborated to include more specific descriptions of behaviour, some of which are more applicable to adults.

(4) The age of onset criteria has been broadened to include 'noticeable inattentive or hyperactive-impulsive symptoms by the age of 12 years'.

(5) The requirement for clear evidence of impairment from the symptoms is a key part of the diagnostic criteria, but is no longer required before the age of 12 years or younger.

(6) Autism spectrum disorder is no longer listed as an exclusion criterion.

These changes recognise that impairment from the symptoms of ADHD may develop later in life and that in some cases symptoms cannot be clearly identified until the early adolescent years. The reduction in the symptom threshold for adults as compared to children recognises the age-dependent changes in the course of the disorder, since the lower threshold in adults is still clinically significant where there is clear evidence of impairment from the symptoms of ADHD; and better reflects the characteristics and natural course of the disorder. These changes mean that many people who previously met the 'in partial remission criteria' will meet full criteria for ADHD under the revised DSM-V (see below for further discussion).

### Clinical presentation in adults

Whereas the core symptoms of hyperactivity, impulsivity and inattention, are well characterised in children, these symptoms may have different and more subtle expressions in adult life. Comparison with the normal behaviour of age, gender and cognitive ability matched groups must be taken into account. For instance, where children with ADHD may run and climb excessively, or have difficulty in playing or engaging quietly in leisure activities, adults with ADHD are more likely to experience inner restlessness, inability to relax, or over talkativeness. Hyperactivity may also be expressed as excessive fidgeting, the inability to sit still for long in situations when sitting is expected (at the table, in the movie, in church or at symposia), or being on the go all the time. Impulsivity may be expressed as impatience, acting without thinking, spending impulsively, starting new jobs and relationships on impulse, and sensation seeking behaviours. Inattention often presents as distractibility, disorganization, being late, being bored, need for variation, difficulty making decisions, lack of overview, and sensitivity to stress. In addition, many adults with ADHD experience lifetime mood lability with frequent highs and lows, and short-fuse temper outburst [35,37,116-118]. Typically, adults with ADHD will not settle after the age of 30 but continue to change and/or lose jobs and relationships, either through boredom or being fired. They are usually underachievers with an estimated annual twenty two days of excess lost role performance [119-122]. As a consequence relationships and jobs are often short lived. Relationships that last are often impaired due to the inability to listen with concentration to the spouse, not finishing or procrastinating tasks, often being on a 'short fuse' and interrupting conversations [123]. Driving accidents are increased in young adults with ADHD as a result of being distracted, impulsive and having an increased need for stimulation [124-127]. ADHD patients are also more likely to be subjected to other accidents like dog-bites and burns, and display an unhealthy lifestyle: smoking, alcohol and drug abuse, riskier sexual lifestyle, a delayed rhythm due to chronic sleep problems, lack of structure and inappropriate healthcare [128-132]. Criminality in adulthood is predicted by ADHD and comorbid conduct disorder in childhood, especially with substance abuse and antisocial personality disorder in adulthood. Among male prisoners, ADHD was found to be strongly related to the number of critical incidents involving aggression and poorly controlled behaviour even after controlling for the presence of antisocial personality disorder [133]. ADHD patients are significantly more arrested, convicted, and incarcerated compared to normal controls, and ADHD is increasingly diagnosed in adults in forensic psychiatry [134-137].

An additional burden on family life may be the presence of one or more children with ADHD, which happens frequently due to the high familial risks of the disorder. Adults with ADHD are at risk for poorer parent-child relationships [138]. Inattention problems often lead to inability to complete independent academic work, resulting in underachievement at school, during study and in the workplace compared to their peer group with equivalent cognitive ability. Therefore many have lower financial resources [17,18,139-141]. Many feel isolated and lonely due to social handicaps and shame about failures. They may have less success with personal growth, lower ability to present themselves in a socially appropriate fashion and lower mental and physical well-being, even in the presence of a high IQ [8,121,142-144].

The clinical picture of ADHD is also coloured by frequent co-morbidity. In childhood, as many as 65% of children with ADHD have one or more co-morbid conditions, including oppositional defiant and conduct disorder, anxiety and mood disorders, tics or Tourette syndrome, learning disorders and pervasive developmental disorders (e.g. autism) [145-147]. Similarly in adults, co-morbidity is the rule, with 75% of clinical patients having at least one other disorder, and the mean number of psychiatric comorbidities being three [41,148]. Mood, anxiety, sleep, personality and substance use disorders are found, as well as learning and other neurodevelopmental disorders [22,37,41,121,144,148-152]. ADHD has also been associated with earlier onset of substance abuse [153]. In adults with ADHD, gambling and other addictions are very common [27,154-158].

### Gender issues

As boys dominate clinical samples of ADHD in childhood, female manifestations and gender differences have been relatively neglected in research as well as clinical practice [159-162]. In childhood ADHD is identified far more frequently in boys than girls with around a one in five ratio in most studies. However, the differences in prevalence and diagnostic rates according to gender become far less skewed with age, as more females are identified and become diagnosed in adulthood [17,161,163,164]; and in some adult clinical series female cases may predominate.

Several factors may explain these observations. In childhood, girls may have less externalizing problems than boys: they suffer more from internalizing problems, chronic fatigue and inattention while boys may be more hyperactive and more aggressive [165,166]. Girls show lower rates of hyperactivity and comorbid conduct disorder than males, and more frequently have the inattentive subtype of ADHD, with a later onset of impairment [162,167]. For these reasons, general practitioners and health care professionals are less aware of ADHD in girls and they are thus less likely to be referred for treatment [168]. In adulthood, the higher prevalence of anxiety and depressive disorders in women may conceal underlying ADHD and influence diagnosis and treatment. As more women seek help from psychiatrists than men, the change in referral pattern may also contribute to the change in gender ratio within clinical populations [169].

## How can ADHD in adults be properly diagnosed?

### Application of clinical criteria

Assessment starts with self-reported symptoms. The physician should perform an in depth diagnostic interview to look for the characteristic psychopathology by careful questioning about childhood and current behavioural symptoms. Although the patient appears to be the best informant, comparison with parent and partner reports in order to provide more information on severity and pervasiveness of symptoms, is desirable [170]. Both DSM-IV and ICD-10 criteria recognise that symptoms of ADHD and HKD persist beyond childhood into adulthood, yet neither set of criteria takes account of age-dependent changes in terms of the number and severity of symptoms, or changes in the way that the symptoms of ADHD might present in adults. Rather, they stipulate the exact same criteria as that applied in children. However developmental changes occur that include an increasing role for inattention and other 'executive function' difficulties in the impairments related to the demands of adult life [171]. The DSM-IV criteria suggest that adults who demonstrate only some of the symptoms of ADHD should be given a diagnosis of "ADHD, in partial remission". This diagnosis, however, seems to underplay the significance of impairments seen in adults who no longer meeting the full DSM-IV criteria but show persistence of some impairing symptoms from childhood. In other words adults seem to outgrow the criteria rather than the disorder.

We therefore conclude that a definition of remission based on the number of criteria that have to be met in childhood does not seem appropriate in clinical practice with adults, especially as symptoms described by both DSM-IV and ICD-10 criteria typically apply to children and are not adjusted for developmental age [13,35,172,173]. Furthermore, restriction of the diagnosis to ICD-10 criteria that focused on a severe form of the combined subtype during childhood will lead to underdiagnosis of adults impaired by ADHD symptoms, especially in women. Symptoms of ADHD in adults should therefore be judged with reference to developmentally appropriate norms. Preliminary research suggests that using the current DSM-IV criteria that stipulate six of nine symptoms of either inattention or hyperactivity-impulsivity, a lower threshold of four of nine of these symptoms in either domain is sufficient to identify impairing levels of ADHD symptoms in adults [17,174]. We therefore recommend that future criteria for ADHD are appropriately adjusted by taking into account age-related changes to symptoms and their relationship to impairment.

Further work is now required to clarify whether broadening the criteria to include those with four or more symptoms in either domain is sufficiently specific within adult mental health populations, since by lowering the threshold other acute psychiatric disorders may generate ADHD-like symptoms that reach this lower threshold. This is however mainly a problem for cross-sectional screening for ADHD, since in clinical practice the diagnosis is based on eliciting a history of ADHD symptoms that start in childhood or early adolescence and are persistent (trait-like) and impairing over time and can therefore be differentiated from adult onset disorders. The other approach under investigation is the identification of alternative descriptions of behavioural impairments seen in adults with ADHD, such as the ecological executive function deficits described by several authors in recent years [175,176], which may show greater sensitivity and specificity for the diagnosis in adults.

As discussed, the expression of ADHD in adults is to some extent different from that in children and the diagnostic descriptions of some of the features need to be adapted to adult expressions of the disorder. For example, physical overactivity in children could be replaced in adulthood by constant mental activity, feelings of restlessness and difficulty engaging in sedentary activities. Furthermore a range of characteristics closely associated with the core ADHD syndrome often lead to some of the impairments typically seen in adults with ADHD. These include symptoms such as disorganization, short-fuse, temper, mood lability and sensitivity to stress [35,116]. Assessment of symptoms is further complicated by the fact that adults have more ways to adapt and/or compensate for problems with attention, hyperactivity and impulsivity. For example adults with high IQ, high socio-economic status or lower levels of comorbid disorders may have better compensatory strategies.

Another difference in the evaluation of ADHD in adults is the usual reliance on self-report of symptoms rather than informant accounts of behaviour by parents and teachers. Although the validity of a retrospective diagnosis of ADHD in adults may be questioned in some cases, this is also true for other psychiatric diagnoses that are dependent on descriptions of current and past behaviour. Self-reports of past and current symptoms can however be reliable if the patient has good insight into the condition [177]. Nevertheless, caution is needed as retrospective recall of childhood symptoms may be compromised in adults with ADHD, with under reporting found to be a common problem due to difficulties with accurate recall [9,178]. While a diagnosis based only on self-report is possible, such an approach may lead to underdiagnosis of ADHD and it may be more reliable to use information from informants as well [179]. Reliance on self-report alone may also risk over diagnosis in some cases, but until now, no evidence has emerged that this is the case [180]. For these reasons it is recommended that whenever possible corroborating information is obtained from a living parent of older relative for childhood behaviour and a partner, relative or close friend for current behaviour and symptoms.

Another important criterion to evaluate is the age of onset. The DSM-IV criterion that some symptoms and impairment should be evident before the age of 7 years is difficult to assess accurately in a retrospective diagnostic assessment in adults. In fact, research has failed to validate to this criterion since it has been found that the clinical syndrome that defines ADHD has similar clinical predictions in terms of course, treatment response and associated impairments regardless of whether symptoms with impairment started before the age of 7 years or later [181-187]. This may be particularly important in relation to the inattentive type which is characterised by a later onset of impairment [172,174,188], as the inattentive symptoms may have been missed in the pre-school years and impairment may not have been noticed or reported until the age of secondary school.

A further problem is recall bias by parents, since after decades they may not accurately recall when symptoms or impairments started. Recent evidence found that on average parents typically report a later age of onset by around 5-years, even where earlier age of onset was known from previous health care records [170,189,190]. One approach to circumvent these problems is to refer to school reports which may provide more accurate and relevant information with respect to the age of onset.

For these reasons it is proposed to use a broader onset criterion, up to early to mid adolescence [186,191] and this approach has been recommended in the recent National Institute of Health and Clinical Excellence in the UK [32]. Clinical judgement should be used in making the diagnosis if symptoms before the age of 7-years cannot be recalled. In most cases a clear report of at least some symptoms associated with impairment is expected by early to mid-adolescence. However, even if there is no clear recall of childhood symptoms, ADHD should be considered when the typical syndrome that defines ADHD is present and there is evidence of lifetime persistence of symptoms indicative of ADHD and associated impairments. This may for instance, be the case in some inattentive but talented people in whom high general cognitive ability and a structured environment may have helped them to cope during childhood and adolescence. The problem of inattention may remain unrecognised until they tried to live independently from their parents and were faced with the organisational and attentional demands of higher education or employment [192].

In addition to the evaluation of symptoms, another important criterion for the clinical diagnosis of ADHD is the presence of significant levels of impairment associated with the symptoms. This is critical because the symptoms of ADHD are found to be continuously distributed throughout the population and there is no natural boundary between affected and unaffected individuals [193]. As with symptoms of anxiety and depression, ADHD symptoms are experienced by most people at times. The disorder is therefore distinguished from the normal range by the severity (extreme nature) and persistence of symptoms, and their association with significant levels of impairment and risk for the development of co-occurring disorders. Criteria relate in part to cultural expectations and this may explain why diagnostic and treatment rates of ADHD are higher in the US than many European countries. From a mental health perspective it is however important to define the impairments from ADHD at a level that most people would consider needs some form of medical, psychological or educational intervention and represents a mental health problem [32]. Furthermore, the diagnosis should not be applied to justify the use of stimulant medication to enhance performance in the absence of a wider range of significant impairments indicating a mental health disorder. Impairments include problems with the following: self-esteem, personal distress from the symptoms, social interactions and relationships, behavioural problems, and the development of comorbid psychiatric syndromes.

Current evidence clearly defines ADHD as a clinical syndrome associated with impairments in multiple domains including academic difficulties, impaired family relationships, social difficulties and increased rates of conduct problems. In adults with ADHD increased rates of antisocial, drug use, mood and anxiety disorders are reported in both cross-sectional and longitudinal follow-up studies; in addition to increased rates of unemployment, poor work performance, lower educational performance, increased rates of traffic violations and accidents and criminal convictions: reviewed in NICE, [32].

Finally, the high familial risk among first degree relatives, in the order of 20% or more [20,194], means that a strong predictor for the ADHD in adults is having a parent, sibling or child with ADHD. Family history of ADHD should therefore raise the index of suspicion and provides further supportive evidence when evaluating individuals for ADHD.

### The assessment process

Diagnosis is based on a careful and systematic assessment of a lifetime history of symptoms and impairment. It is not just based on a single clinical impression gained during consultation. Central to this process is the assessment of childhood-onset, current symptoms of ADHD and the presence of symptoms and impairment in at least two domains (school, work, home, interpersonal contacts). Associated features should be evaluated including mood lability, temper outbursts and comorbid disorders (see differential diagnosis). It is important to take a full medical history of psychiatric and somatic treatments, as well as a family history of psychiatric and neurological problems. It is useful to ask the patient about the pattern of symptoms, typical of ADHD and its co-morbidities in his/her family, taking into account familial factors and the high heritability of the symptoms. Patterns of comorbidity in children and adults with ADHD have been identified and include: mood, anxiety, sleep, conduct and substance use disorders as well as personality disorders. Some care must be taken to distinguish between symptoms that often co-occur with the core syndrome of ADHD (e.g. mood instability, ceaseless mental activity, avoiding situations such as waiting in lines when frustration may occur) from those of a separate comorbid condition (e.g. bipolar disorder, major depression, anxiety, personality disorder). Comorbidity being the rule rather than the exception, the evaluation of co-occurring symptoms, syndromes and disorders must always be part of the clinical assessment of adult ADHD [148,150].

ADHD is also associated with increased rates of neurodevelopmental traits and disorders including autism spectrum disorder [195], dyslexia [196] and impaired motor coordination [197]; which are thought to arise from overlapping genetic influences. Such neurodevelopmental comorbidities are less well studied in adults with ADHD, but they are commonly observed in clinical practice and may lead to continued impairments following successful treatment of ADHD symptoms with medication. It has also been the practice in some European countries to look for soft neurological signs during the assessment of adult ADHD, in analogy with the diagnosis of DAMP in childhood (Deficits in Attention, Motor control and Perception), that often accompanies ADHD [198,199]. However there is limited data on the DAMP syndrome in adults.

Comorbid substance use disorder (SUD) deserves special attention due to the high rates of ADHD within SUD populations. A bidirectional link between ADHD and SUD is reported [142,200] with ADHD symptoms over represented in SUD populations [201] and SUD in ADHD populations [202]. From twenty five published studies that screened for substance misuse in ADHD samples prevalence was estimated to be around 45% to 55%. Alcohol and cannabis are the most frequently abused substances in these populations [201] followed by lower rates of cocaine and amphetamine abuse [203]. In contrast, from ten studies that screened for undiagnosed ADHD in SUD populations estimates of ADHD ranged from 11% [204] to 54% [205]. The causes for such comorbidity are likely to be complex including altered reward processing in ADHD, increased exposure to psychosocial risk factors and self treatment. Sometimes patients with ADHD describe self-treatment with unprescribed stimulants, such as parents trying their child's medication. In some other cases, paradoxical reactions to drugs of abuse are reported by patients who feel calmer, better able to concentrate and are less impulsive. While consuming drugs with a stimulant action such as cocaine or amphetamine is described in a few cases, it is more common for adults to describe a general reduction in symptoms from alcohol and cannabis [206,207].

Although there are many challenges in identifying undiagnosed ADHD in SUD settings, systematic screening is feasible [208]. This is particularly important since SUD patients with comorbid ADHD often present with severe forms of SUD [209] characterised by early onset, extended duration of SUD, greater impairment and a shorter transition from substance use to dependence [172]. ADHD has been found to increase suicide risk in SUD adolescents [210]. In SUD treatment outcomes, methadone maintenance patients in the USA with significant ADHD symptoms in the two weeks prior to admission were less likely to achieve abstinence [211].

Close supervision is advised when treating ADHD patients with SUD with stimulants and in cases where diversion or abuse is a particular concern, atomoxetine may be selected as the first line treatment. Research in ADHD populations suggests that only a small minority divert or misuse their medication [212] and certain long acting formulations with a low abuse potential can be used, although studies of adolescents suggest that 75% of patients who do misuse medication have comorbid SUDs [213].

### Instruments for screening and diagnosis

There are many screening instruments and diagnostic interviews available, some of which have been translated into different languages. Commonly used rating scales for screening include the ADHD Rating Scale, based on the DSM-IV criteria [214], and the six item World Health Organisation Adult ADHD Self-Report Scale (ASRS) Symptom Checklist (online available without limitation and in many different languages at http://www.hcp.med.harvard.edu/ncs/asrs.php[215]. The ASRS includes questions for each of the 18 DSM-IV items, re-worded to better represent the presentation of the ADHD items in adults. The 6-item short version was selected on the basis of sequential logistic regression, to optimize concordance with the clinical classification; and was estimated to have a sensitivity of 68.7% and specificity of 99.5% with total classification accuracy of 97.9%, evaluated using population survey data [216]. Specificity of this and other screening tools may be lower within clinical samples with high rates of other mental health disorders; and positive screens should always be followed by full diagnostic evaluations based on clinical interview data. The ASRS, for example, has been investigated in a sample with substance use disorders and the sensitivity found to be higher (87.5%) and specificity lower (68.6%) than that reported in the previous study [217].

To guide the diagnostic assessment process, other self-report questionnaires are available, such as the Brown ADD Scale Diagnostic Form (BADDS) [39] that measures only behaviours relating to executive functioning and inattention; the Conners' Adult ADHD Rating Scale that includes the DSM-IV criteria and has different versions for patients and for significant others (CAARS) [218]; and the Wender Utah Rating Scale (WURS) [219] that includes also symptoms of other, often comorbid disorders [220]. The total symptom scores of the ADHD Rating Scale and the CAARS may be used to screen for ADHD and to evaluate treatment outcome.

For the main diagnostic assessment use of a structured diagnostic interview is advised, such as the Conners Adult ADHD Diagnostic Interview for DSM-IV (CAADID) [221]. An alternative under development is the Diagnostic Interview for ADHD in adults (DIVA) [222]. Although several of the rating scales and interviews are available in different languages there is a need for validating these translations for use throughout Europe.

Currently there are no neurobiological or neuropsychological tests for ADHD with sufficient sensitivity and specificity to serve as an individual diagnostic test [223]. Functional imaging seems promising although more research is needed to establish its value [224-226]. Neuropsychological tests (e.g. the CANTAB tests, the Stop Signal Reaction Time, IQ or computerized tests of executive functions and speeded reaction time responses) may complement diagnostic assessments and can provide an objective index of cognitive functions in the patient with ADHD [227]. There is a recent surge in interest in the use of cognitive-electrophysiology that may provide data that is more sensitive to the diagnosis than cognitive-performance data alone [228,229]. Patients with ADHD may suffer additional cognitive deficits, which may contribute to functional impairment, such as in learning, reading, writing difficulties, in addition to impairments related to the autistic traits. As there are no established norms for learning disorders in adults, it may be difficult to distinguish these disorders from ADHD-related learning deficits. Further research is required to provide a full understanding of the cognitive impairments associated with ADHD in adults.

Despite the poor predictive value of cognitive performance tests, some experts conceptualise ADHD as primarily a deficit of executive functions. While this may not always be seen in cognitive performance deficits, impairments are usually seen in the way that people with ADHD manage daily tasks [175,176]. Many problems reported by adults with ADHD are thought to reflect executive dysfunctions, including impaired self-organisation, attentional and emotional regulation, sustained effort and alertness; that may only been seen in performance deficits in every day life and not under test conditions. This has led to efforts to provide more sensitive behavioural descriptions of performance deficits seen in ADHD that in general reflect problems of self-control and self-regulation [230]. These new studies are likely to lead to improved and more sensitive symptom and behavioural checklists for ADHD in adults.

### Differential diagnosis

It is important for the diagnosis of ADHD, as well as the correct targeting of treatments, to identify comorbid conditions such as mood, anxiety, psychotic, organic and substance use disorders; in addition to personality, tic and autistic spectrum disorders. Because adults with ADHD often exhibit low self-esteem, low mood, affective lability and irritability, these symptoms may sometimes be confused with dysthymia, cyclothymia or bipolar disorder and with borderline personality disorder. Furthermore daily mood changes in ADHD are very common, and represent a poorly regulated but essentially normal range of moods, rather than the more severe extremes of depression and elation seen in bipolar disorder; and it is argued that chronic mood instability should be considered part of the core syndrome of ADHD [118,175,231].

ADHD and borderline personality disorder seem to share impulsivity, affective instability, anger outbursts and feelings of boredom [35,117,218]. In the ADHD patient, impulsivity and anger is usually short-lived and thoughtless, rather than driven; conflict relationships, suicidal preoccupation, self-mutilation, identity disturbances and feelings of abandonment are usually less intense than in borderline personality disorder. However, the differences may not be clear-cut because in both disorders symptoms are chronic and trait-like. Importantly, individuals presenting with a diagnosis of personality disorder who present with the ADHD syndrome that started in childhood will in many cases benefit from pharmacological treatments for ADHD [232,233]. Recent trials in ADHD patients indicate that besides ADHD symptoms, mood instability improves with both stimulant treatment and atomoxetine [234,235]. As the order of treatment will depend on the presence of and severity of co-morbidities, evaluation of co-morbid disorders is a key component of the ADHD assessment using appropriate clinical diagnostic approaches.

## How to treat adults with ADHD

### Effective treatments

The symptoms of ADHD can be treated effectively in both children and adults. The beneficial effects of stimulant medication and atomoxetine on the core symptoms of ADHD have been demonstrated in numerous studies in children. An increasing number of studies in adults demonstrate a similar clinical response to that seen in children [32,236-239]. Due to the demands and responsibilities of adult life, adults face many problems that are different from those faced by children and they therefore need a different range of psychosocial and psychological treatments tailored to both their developmental level and ADHD. Psychological treatments in the form of psychoeducation, cognitive behaviour therapy, supportive coaching or assistance with organising daily activities are all thought to be effective [240-242]. Further research is however needed as there is an insufficient evidence base to recommend their routine use in clinical practice [32].

### Impact of non-treatment

Long-term follow-up, epidemiological and clinical studies have shown that adults with untreated ADHD, when compared to normal controls, experience higher rates of academic failure, low occupational status, increased risk of substance use disorders (tobacco, alcohol or drugs), accidents and delinquency, and have fewer social relationships or friends [18,121,127,131,243,244]. Patients diagnosed with ADHD in adulthood often complain that they did not receive treatment earlier in life and feel that their life would have been different if they had. Appropriate treatment could have prevented accidents and ongoing impairment at school, at work and in their peer and partner relationships. After decades of undiagnosed and unmet needs, the diagnosis offers an explanation for their problems which is valuable to a lot of patients and their family. Treatment of adult ADHD can influence psychosocial impairment that results as a consequence of 'core' ADHD symptoms, and may lead to improvements in associated features and comorbid disorders [245-247]. These include the following:

• psychological functioning and self-confidence

• family/relationship functioning

• interpersonal (broader than family) functioning

• professional/academic functioning

• cognitive deficits

• driving performance

• risk of substance use disorders

### Optimal treatment algorithm

Similar to the treatment of ADHD in children, a multimodal approach to treatment of adults with ADHD and associated co-morbid disorders should be taken [248]. Ideally, the treatment plan would also involve the adult's partner, family or close relationships. The multimodal approach includes:

• psycho-education of ADHD and comorbid disorders

• pharmacotherapy for ADHD and comorbid disorders

• coaching

• cognitive behaviour psychotherapy (individual and group)

• family therapy

### Treatment focus in co-morbid ADHD

Treatment should follow careful diagnostic assessment of ADHD and associated comorbid disorders. In the case of comorbidity, the integrated treatment plan should address both ADHD and the comorbid condition, the order of pharmacological treatment depending on the type and severity of comorbidity. Generally, severe mental health disorders should be treated first, such as in-patients with psychosis, major depression, mania or drug addiction; following which the diagnosis of ADHD and need for treatment can be reviewed. However, treatment of milder depressive and anxiety disorders may be deferred until after treatment of ADHD and often needs no further treatment as the comorbid symptoms may resolve following effective treatment of ADHD. Symptoms such as demoralisation and low self-esteem following a life with ADHD, and mood instability, improve with stimulant treatment alone [234]. It is up to the clinician to decide on what is the most important need for every patient with specialist advice being sort for more complex cases.

With respect to substance use disorders, ADHD is considered an important factor in its aetiology as the substances are often used for self-medication and may relieve symptoms like restlessness, inattention, impulsivity and sleep problems [207,249-251]; or may be taken as part of stimulus seeking (novelty seeking) traits that are also associated with ADHD. In such cases, treatment of ADHD may help patients to stop substance use for self-medication [252-254] or may reduce impulsive stimulus seeking behaviour. However, systematic research has not provided a strong evidence base for appreciable improvements in ADHD when treated in the presence of substance use disorders [255,256]; and drug or alcohol abuse disorders should always be targeted as a primary disorder. Treating ADHD in parallel with SUD can however be important in some cases, particularly where ADHD is severe or where there is good understanding and compliance for the treatment program.

However, in some countries, regulatory rules will not allow prescription of stimulants in patients with substance use disorders. Patients should be asked to register their drug/substance intake and be encouraged to stop their use. Despite concerns that pharmacotherapy with stimulant medication may be a risk factor for substance abuse, the literature supports the view that stimulant treatment for ADHD either has no impact in risk for substance abuse, or may even lower the risk of substance abuse by reducing the early onset of substance abuse in adolescents [206,254,257-259].

### Psycho-education

Psycho-education is the first step in the treatment plan and involves educating the patient and ideally also the partner or family about ADHD symptoms and impairment, the prevalence in children and adults, the frequent comorbidity, the heritability, the brain dysfunctions involved, as well as the treatment options. In many cases, simply providing the patient with this information may help the patient's understanding and bring him or her comfort. Often this process offers new insights into past difficulties. Relationship difficulties often decline after this sharing of information with family members. Feelings of guilt and remorse can be left behind, and the patient's social network may begin to be restored. This social network will prove invaluable to the patient during the treatment process. Informing the patient about the existence of self-help groups may be valuable, allowing him or her to join and share information and experiences, as well as to gain further comfort and understanding. There is a need to further develop structured psychoeducation programs with specific objectives.

### Pharmacotherapy for adult ADHD in Europe

Stimulants (methylphenidate and dexamphetamine) are first choice medication treatments for ADHD in children and adults, based on an extensive and still growing body of data concerning efficacy and safety [32,260]. Atomoxetine is usually considered the second line treatment, followed by other non-stimulants like bupropion, guanfacine, modafinil and tricyclic antidepressents, based on efficacy outcomes in controlled studies in different age groups [239,261-264].

Stimulants are effective in about 70% of patients with ADHD in controlled studies [265-267] depending on the study design and maximum drug dosage. A recent European study of adults with ADHD showed the effectiveness of methylphenidate over a period of six months, in the longest double blind placebo controlled trial to date [268]. Stimulant treatment not only improves the symptoms and impairing behaviours associated with ADHD, but also improves related problems such as low self-esteem, anger outbursts, mood swings, cognitive problems and social and family function. Side-effects are usually mild and transitory, mainly consisting of headache, reduced appetite, palpitations, nervousness, difficulty falling asleep, and dry mouth [148,269,270]. Stimulants may increase blood pressure and heart rate, and decrease weight, therefore patients should be assessed regarding these issues prior to, and monitored during treatment. Stimulants are not advised during pregnancy or breastfeeding and are contraindicated in psychotic disorders up to now; although some specialists have treated ADHD successfully in stable patients with schizophrenia maintained on antipsychotics [271]. Relative contra-indications are hypertension, cardiac problems including angina, hypertrophic cardiomyopathy and arrhythmias, hyperthyroidism and glaucoma. For these disorders, first referral to and treatment by a specialist are needed before starting a stimulant. Stimulants may be used in autism with generally positive effects, although in some cases autistic features may worsen [272-275]. A recent meta-analysis indicated that treating ADHD in children with tic disorders is safe and effective [276,277]. Stimulants have little impact on seizure threshold and may be used in epilepsy [278].

Although stimulants are by far the best studied and most effective treatment for ADHD, their use in some parts of Europe is still controversial in both children and adults. It is not yet common practice for doctors to prescribe stimulant medication to adults. As a result support services and expertise for treating ADHD in adults are not always available. As discussed earlier, the use of stimulants continues to be hampered by stigma and lack of up to date information in their use, with restricted access in many European countries [279].

The hesitancy and uncertainty about the appropriate use stimulant medication by both the general public and many physicians may also be related to the history of abuse with amphetamines in the past. Health authorities have given methylphenidate the same drug classification as amphetamines and they both perform similar to each other in traditional assays of abuse [280,281]. Abuse potential has been studied by Nora Volkow (Director of the National Institute of Drug Abuse, USA) who showed that abuse potential relates to the route of administration, with rapid rise in dopamine levels following injection or snorting of stimulants. In contrast, during oral clinical use, methylphenidate elicits slow steady-state dopamine increases in the brain which mimic those of tonic neuron firing, rather than rapid dopamine changes associated with reinforcing drug-like effects [257]. In the Volkow study it was found that intravenous methylphenidate could not be distinguished from cocaine by cocaine addicts, whereas this was not the case for oral methylphenidate. Therefore the use of extended release medications that have a slow rate of serum rise, are preferable to reduce potential for abuse and diversion.

Importantly, both clinical studies and clinical experience support the view that the methylphenidate does not lead to stimulant or drug addictions. On the contrary, it has been shown to have a neutral or reducing impact on substance abuse and the risk of relapse [206,253,254]. Furthermore, stimulants are not addictive from the clinical perspective. Adolescents treated from childhood generally use less stimulant medication or stop, using instead of taking more. A common problem is poor compliance or cessation of treatment during the adolescent years. There is also no clear evidence of tolerance over time. Neither has stimulant use been associated with adverse effects on driving, but rather is associated with an improvement in the concentration of ADHD patients during driving [127,282]. The main potential problem associated with their use and reported in a number of studies from the US is the inappropriate diversion of stimulants, by parents self-treating themselves with their children's medication or as a cognitive enhancement medication for college students. This should not however detract from their specific use to reduce ADHD symptoms and associated impairments in people with ADHD [283].

Continued research in adult patients and education about efficacy and safety may help to overcome these problems. The non stimulant atomoxetine may be an alternative to treatment with stimulants in substance abuse patients with ADHD, although studies showing superiority over stimulants in this difficult patient population are still lacking [200,284-286].

### Types of stimulants

In the United States, more than ten different stimulant preparations have been developed for treatment of ADHD, the most recent being long acting formulations of oros-methylphenidate, mixed amphetamine salts, dexmethylphenidate and lis-dexamphetamine [265,287,288]. These improvements were necessary because of the very short half-life leading to relatively short duration of symptom control from immediate release methylphenidate (two to four hours) and dexamphetamine (three to five hours). The requirement for a longer duration of activity in adults, requires repetitive dosing with immediate release stimulants, of between three to four doses in most cases, and more often in others, to avoid rebound symptoms and for adequate control of ADHD symptoms during the day and evening [148,270]. Compliance to such frequent dosing regimens is however poor in ADHD patients due to forgetfulness, inattentiveness and self-organisation problems, leading to daily instability by frequent rebound symptoms and ineffective treatment [148,289-291].

Extended release preparations of stimulants have durations of action between 6 to 14 hours, which may permit once-daily dosing, although an adult may still need twice daily dosing of medication for a 12-16 hour day. Currently, combinations of immediate and extended release preparations are often prescribed in adults with the aim of tailoring the dose regime to the individual requirement and medication response of each patient. Dosing schemes and maximum daily dose vary across Europe (from 0.3-1.5 mg of methylphenidate/kg/day). However, the panel recommends that the dose of stimulants in adults should be individually adjusted, based on response and tolerability. The short half life of the medications suggest that it is more practical to consider maximum dose in terms of the maximum taken at each time point, the length of the effect of each dose on the control of ADHD symptoms, and the number of doses required to provide symptom control throughout the day; rather than a maximum based only on mg/kg per day. Individual differences in the optimal dose response to stimulant medication means that the best approach is to titrate the dose for each individual, starting at a low dose and increasing to an effective dose, while keeping side effects to a minimum; and should not be determined on a mg/kg basis.

In Europe, limited and different products are registered in the various countries. Some countries still only have access to immediate release methylphenidate and may or may not have access to dexamphetamine, while increasingly the longer-acting extended release preparations are being introduced. In Switzerland Dexmethylphenidate XR was licensed for use in adults in 2009 and this has been reported as a safe and effective option [292].

### Second line pharmacotherapeutic treatments

For adults with ADHD who do not respond to stimulant therapy or who have a condition in which a stimulant is contraindicated, the non-stimulant atomoxetine that is licensed for child and adult ADHD in the USA is an appropriate alternative [247]. Atomoxetine has an effect size of around 0.4 in adults [32,293], a duration of action of 24 hours, and no abuse potential [294]. Atomoxetine may be indicated in patients with comorbid substance use disorders, emotional dysregulation or social anxiety [295-297]. Other choices comprise medications like long acting bupropion, modafinil and guanfacine, that have all been investigated in ADHD [262,298-300]. Tricyclic antidepressants like Desipramine, an imipramine metabolite, has been shown to be effective in adults with ADHD [301]. However, these medications must be considered fourth line agents due to their side effects, limited value in treating the symptoms of inattention and relatively low effect size compared to stimulants in the treatment of ADHD [302].

In more complicated co-morbid cases, clinical experience indicates that treatment may be combined with antidepressants and mood stabilizers, although controlled studies are still lacking [303]. A recent review on drug interactions in the treatment of ADHD concluded that methylphenidate appears to be more implicated in pharmacokinetic interactions suggestive of possible metabolic inhibition, while amphetamine was more involved in pharmacodynamic interactions and could potentially be influenced by medications affecting cytochrome P450 (CYP2D6) [304]. Only monoamine oxidase inhibitors (MAOIs) are contradicted with the concomitant use of stimulants. Other drugs such as antidepressants and antipsychotics can be given at the same time as stimulants but in a few cases some adjustment in the dose of either drug might be required. Finally, there is no consistent evidence from randomised control trials for the use of food supplements, such as omega-3 fatty acids ADHD [305].

### Coaching and Cognitive Behavioural Therapy

Pharmacotherapy alone is usually not sufficient to stabilise the many problems of adults with ADHD. Coaching provides a structured, supportive therapy, either individually or in group sessions. Coaching aims to teach problem-solving skills for identified practical problems. Due to a lifetime history of the impairment, adults with ADHD have typically not learnt practical organisational skills [306,307] and may have developed poor coping skills and inappropriate behaviours in response to the impairments associated with ADHD.

A coaching program may include:

• acceptance of the disorder

• learning to deal with time management

• learning to limit activities to 'one goal at a time'

• organising home, administration, finances

• dealing with relationship and work difficulties

• learning to initiate and complete tasks

• understanding emotional responses associated with ADHD

These components of coaching are also addressed by cognitive behavioural therapy for adult ADHD [241,308]. According to the clinical experience of the panel, many adults may benefit from supportive and/or cognitive behavioural therapy in combination with pharmacotherapy. Other forms of psycho-social or family therapy may help with impairments associated with ADHD, such as relationship problems and low self-esteem [309]. Psychotherapy targets the adaptation of the ADHD patient to a lifelong debilitating disorder and it may relieve co-morbid symptoms.

Current research does not support the efficacy of psychotherapeutic treatments as sole treatment for adult ADHD; neither do they relieve the core symptoms of ADHD. They are however considered an import adjunctive treatment for people who prefer a psychological approach or where residual symptoms or comorbidities remain. CBT has a strong evidence base for the treatment of some of the comorbidities associated with ADHD yet there are few controlled trials in adults with ADHD. The findings in ADHD are reviewed by the Research Forum on Psychological Treatments for Adults with ADHD, who identified moderate to large effect sizes from five empirical studies. They concluded that psychological treatments may play a critical role in the management of adults with ADHD who are motivated and developmentally ready to acquire new skills as symptoms remit [310]. Safren and colleagues [241] compared CBT plus medication with medication treatment alone and found significantly greater improvements for ADHD symptoms and anxiety and depression scores in the combined treatment group. Similar findings were reported by Rostain and Ramsey [247], while a further study reported added benefits from CBT and psychoeducation delivered in a brief intensive group format [311]. Alternative strategies have focused on strengthening cortical (executive) function with several studies indicating treatment effects in ADHD symptoms using techniques such as working memory training and neurofeedback and cognitive remediation therapy in children [312-316] and meta-cognitive training in adults [317]. A full review of psychological approaches for ADHD throughout the lifespan has recently be published by Young and colleagues [240]

### Prognosis and costs

ADHD in adults presents as a lifelong condition that started during childhood. The precise details of the clinical presentation may change with age and with the demands of adult life, but are broadly similar to those seen in children. As medication treatment for ADHD does not cure the disorder and some or all symptoms may return after discontinuation of medication, long-term pharmacological and psycho-social treatment may be necessary. The poor long term prognosis of untreated ADHD has implications for the costs of illness. In children, the economic burden of ADHD has been estimated to be approximately double those of normal controls, due to substantially more inpatient as well as outpatient hospitalisations and emergency department visits [318]. The economic burden of untreated adult ADHD has been increasingly studied and shows the same pattern as in children, with higher than normal costs of sickness leave, less productivity, more accidents and more health care costs [141,319,320]. Effective management of the patient with ADHD is justified from a health economic perspective since undiagnosed and untreated ADHD will lead to inefficient health care use, less satisfactory clinical outcomes, lower personal well-being and poorer social and professional interactions.

## Conclusions

A summary of the main conclusions is listed in Table 1. ADHD persists in adults in the majority of subjects with significant psychosocial impairment and a high comorbidity rate leading to high levels of personal distress and a substantial economic burden for society if left unidentified and untreated. The lifetime persistence of symptoms and impairment of ADHD is the hallmark of this disorder in the majority of cases. Diagnosis should include extensive psychiatric work-up including a detailed account of the developmental history, both current and retrospective account of ADHD symptoms and impairment and associated co-morbidities, before starting treatment. To prevent underreporting of symptoms, external validation is desirable by collecting information from relevant informants. The panel recommends multimodal treatment, comprising of psychoeducation, pharmacotherapy, coaching and/or cognitive behavioural therapy; and ideally involving the adult patient's partner, family or close friends. Diagnostic and treatment services for adult ADHD should be established throughout Europe. European research into adult ADHD should be further developed in order to provide a better understanding of the way that ADHD presents from childhood until old age, to differentiate ADHD from common comorbidities and to improve treatment options.

**Table 1:** Summary of key points from the consensus statement

Neurobiological and environmental background	• Anomalies in brain functioning identified in case control studies of cognitive, electrophysiological and neuroimaging studies, and the effectiveness of pharmacological treatments with dopamine agonists support the neurobiological underpinnings of ADHD.
	• High heritability and associated environmental risk factors suggest a primary role for genetic influences that are moderated by environmental factors in the majority of cases.
**Diagnosis**	• The gender differences in child and adult ADHD may be due to different expression of symptoms and co-morbidities, perception of impairments, and referral bias; and deserve further study.
	• Age, gender, and IQ matched reference values are still lacking for diagnostic assessment of ADHD in adults.
	• The age-of-onset criteria required for the retrospective diagnosis of ADHD in adults is less reliable and less important than the persistence of symptoms and impairment of ADHD during the lifespan.
	• A cut off of 4/9 current DSM-IV criteria may be sufficient in adults with a childhood onset of symptoms, when accompanied by significant impairments.
	• Age-appropriate presentations of ADHD symptoms should be taken into account when scoring the symptoms of ADHD in adults.
	• The diagnosis of ADHD in adulthood should be based on self-report and in-depth evaluation, but collateral information is desirable.
	• Various instruments are available for screening and assessment of ADHD, but validation studies are urgently needed.
	• Neurobiological and neuropsychological tests are neither imperative nor sufficient for the diagnosis of ADHD but may document specific functional impairments.

**Treatment**	• Non- treatment may deprive the patient of the chance to resolve functional and psychosocial impairments at personal, relationship and professional levels.
	• The severity of ADHD and associated co-morbid disorders should be the first guide to select which disorder to treat first. Treatments can often be combined.
	• Albeit local regulatory rules may dictate differently, in patients with ADHD and substance use disorder, ADHD treatment with stimulants should not be withheld, but rather postponed until problematic substance use is stopped and there is a commitment to the treatment process.
	• Stimulants are the treatment of choice for adults with ADHD. Long-lasting, extended release formulations are preferred for reasons of adherence to treatment, for the protection against abuse, to avoid rebound symptoms, for safer driving, and to provide cover throughout the day without the need for multiple dosing.
	• The non-stimulant atomoxetine can be a second line treatment. Others like modafinil, bupropion, guanfacine, and tricyclic antidepressants have shown efficacy in controlled studies.
	• Psychotherapy targets the relief of co-morbidities and behavioural, social, cognitive or other functional impairments.
	• The poor long-term prognosis in a substantial part of patients and the absence of a cure upon stopping medication underline the role of long-term management of the adult with ADHD.
	• With appropriate diagnosis and treatment, morbidity may be low, health care use efficient, outcomes better and associated with lower economic burden.
	• More research on ADHD throughout the lifespan until old age is needed.

## Competing interests

The following co-authors have no competing interests: Dr Pierre Oswald, John Fayyad, Doris Ryffel, Karen Foeken, Pieter-Jan Carpentier, Chantal Henry, Martin D. Ohlmeier, Helmut Niederhofer, Stefano Pallanti, A. Pehlivanidis.

The following co-authors have the following competing interests: Dr. J.J.S. Kooij has participated in research with and has been on the speaker bureau of Janssen-Cilag and Eli Lilly. Dr Steven Stes have served as a consultant for Novartis, received educational grants from Janssen-Cilag, conference attendance support and/or served on the speaker bureau of Janssen-Cilag, Novartis, GlaxoSmithKline and Lundbeck, served on the advisory board of Janssen-Cilag and Novartis and he is also a co-author of a book about adult ADHD that is commercially available (for patients and their environment). Dr. J. Antoni Ramos-Quiroga has received fees from Janssen-Cilag, Shire, Lilly and Rubió and funds from Janssen-Cilag, Lilly and Rubió. Dr. Miguel Casas has received fees from Janssen-Cilag, Lilly and Rubió and funds from Janssen-Cilag, Lilly and Rubió. Dr Dan Edvinsson have been a Consultant for Bristol-Myers Squibb and Novartis, a speaker for Janssen-Cilag, Novartis and Lundbeck; and participated in medical trials for Janssen-Cilag (Lambda 1+2). Dr Ylva Ginsberg has served as a consultant and speaker for Janssen-Cilag and Novartis and as a speaker for Lundbeck. She is a Principal Investigator for LAMDA 1 and LAMDA 2 funded by Janssen-Cilag. Dr Michael Fitzgerald has been a consultant for Shire and has been on the advisory board for Lilly and Janssen Cilag. Dr Michael B. Lensing has received honorarium from Janssen Cilag as member of a Scientific Committee in connection with NPA (Nordic Psychiatric Academy ADHD) meetings in 2009 & 2010. Dr Maria Råstam has acted as speaker for Eli Lilly, Jansen Cilag and Novartis. Dr Iris Manor is a consultant for Janssen-Cilag and has been Principal Investigator for Enzymotec Ltd. and Alcobra Ltd. and funded by them for this work. She has also given courses and lectures to Israeli specialists and pharmacists that were funded separately by Janssen-Cilag, Teva and Novartis. She was funded for the IMAGE study by the NIH and is a member of APSARD (The American Professional Society of ADHD and Related Disorders) and was funded for a CME about ADHD. Dr Andrew Blackwell is an employee of Cambridge Cognition Ltd. Dr Hervé CACI have in the past five years received reimbursements and/or fees from Lilly and Shire and also been an investigator or principal investigator in studies conducted by Lilly, Sanofi-Synthélabo, Janssen-Cilag and Shire. Dr Gaillac Veronique took part in the Eli Lilly atomoxetine adult study (LYDO) and a clinical study on adult ADHD sponsored by Bioproject. Philip Asherson has received speaker fees and been on advisory board meetings for Janssen, Shire and Flynn Pharma. He received an educational grant from Janssen and is principal investigator for a study funded by Shire.

## Authors' contributions

We thank all co-authors for their considerable expertise in generating the consensus paper. Authors all gave freely of their time for this work. All authors took part in meetings between 2003 and 2009 which formulated the content of the consensus statement. A formally prepared document with consensus statements was generated by SK and subsequently revised by PA, with the assistance of all co-authors. The entire content was reviewed by all the co-authors. Following this process the final document was circulated for written approval by all members of the European Network and further changes made on the basis of written comments and text changes.

## Pre-publication history

The pre-publication history for this paper can be accessed here:

http://www.biomedcentral.com/1471-244X/10/67/prepub
